# Controlling Cytokine Release Syndrome to Harness the Full Potential of CAR-Based Cellular Therapy

**DOI:** 10.3389/fonc.2019.01529

**Published:** 2020-01-31

**Authors:** Monica S. Thakar, Tyce J. Kearl, Subramaniam Malarkannan

**Affiliations:** ^1^Laboratory of Molecular Immunology and Immunotherapy, Blood Research Institute, Versiti, Milwaukee, WI, United States; ^2^Department of Pediatrics, Medical College of Wisconsin, Milwaukee, WI, United States; ^3^Department of Medicine, Medical College of Wisconsin, Milwaukee, WI, United States; ^4^Department of Microbiology and Immunology, Medical College of Wisconsin, Milwaukee, WI, United States; ^5^Center of Excellence in Prostate Cancer, Medical College of Wisconsin, Milwaukee, WI, United States

**Keywords:** cytokine release syndrome, chimeric antigen receptor, T cells, NK cells, Fyn-ADAP

## Abstract

Chimeric Antigen Receptor (CAR)-based therapies offer a promising, targeted approach to effectively treat relapsed or refractory B cell malignancies. However, the treatment-related toxicity defined as cytokine-release syndrome (CRS) often develops in patients, and if uncontrolled, can be fatal. Grading systems have now been developed to further characterize and objectify clinical findings in order to provide algorithm-based guidance on CRS-related treatment decisions. The pharmacological treatments associated with these algorithms suppress inflammation through a variety of mechanisms and are paramount to improving the safety profile of CAR-based therapies. However, fatalities are still occurring, and there are downsides to these treatments, including the possibility of disrupting CAR-T cell persistence. This review article will describe the clinical presentation and current management of CRS, and through our now deeper understanding of downstream signaling pathways, will provide a molecular framework to formulate new hypotheses regarding clinical applications to contain proinflammatory cytokines responsible for CRS.

## Introduction

Although great progress has been made in treating hematologic malignancies, patients having relapsed or refractory disease often have poor outcomes. In adults, one of the most common hematologic malignancies is diffuse large B-cell lymphoma (DLBCL), which affects an estimated 28,000 patients in the United States each year (1). Up to 50% of patients with DLBCL have refractory disease or experience relapse after initial treatment. For these patients, outcomes are very poor with long term survival rates of 20–53% (2, 3). In the pediatric population, precursor B-cell acute lymphoblastic leukemia (B-ALL) is the most common malignancy. Although >90% of children enter remission and have excellent rates of long-term survival, relapse can occur in approximately 20% of all cases. Those who relapse often succumb to their disease (4–7).

Chimeric antigen receptor (CAR) T-cell based therapies are an exciting, targeted approach to effectively treat relapsed or refractory hematologic malignancies and have helped bring immunotherapy to the forefront of cancer treatment. CAR-T cells are a biological drug that targets cancer-associated antigens using genetically-modified autologous T cells transduced with the CAR (8). Successful outcomes from early clinical trials using CD19-targeted CAR T cells have led to the FDA approval of tisagenlecleucel in 2017 and 2018 for relapsed or refractory pediatric B-ALL and large B cell lymphomas, respectively, and axicabtagene ciloleucel in 2017 for relapsed or refractory large B cell lymphomas. Building off these recent successes, new clinical trials are exploring a variety of ways to expand this therapy, including the development of CAR-based natural killer (NK) cell immunotherapy to work in diseases and circumstances where CAR-T cells may falter (9). Despite high rates of complete and partial remissions, allowing for long-term durable survivals and/or providing bridging therapy until hematopoietic cell transplantation, the trials leading to approval of CAR-T products also demonstrated significant therapy-related toxicities, specifically cytokine release syndrome (CRS) and its accompanying neurological effects (10–13).

Cytokine storm was initially used to describe the systemic inflammatory response that occurred following any antibody-based immune therapy; however, since the incidence of what is now coined CRS following CAR T-cell based therapies is high, the use of this term is now fairly synonymous with the major adverse effect of this therapy (13). CRS can begin with mild symptoms such as fever, tachycardia, tachypnea, nausea, and diarrhea. Concurrently, a spectrum of neurological toxicities known as CRS-related encephalopathy syndrome (CRES) [or immune effector cell-associated neurotoxicity syndrome (ICANS)] can develop, consisting of headaches, confusion, aphasia, and seizures. In its more severe forms, the inflammation related from CRS and/or CRES/ICANS can result in hypotension, coagulopathies, cerebral edema, and multi-organ failure (14–16). If uncontrolled, death can result. Across nine separate studies of CD19 CAR T cells including 387 patients, 83% developed CRS and 51% developed severe (>grade II) CRS (13, 17–24). The ELIANA trial (a phase III trial of tisagenlecleucel for pediatric and young adult patients) illustrates the potential morbidity of CRS. After receiving tisagenlecleucel, 77% of patients developed CRS, 25% required high-dose vasopressors for cardiac support, 13% were intubated, and 9% required dialysis (17).

Numerous CAR-based clinical trials are underway to treat hematological malignancies and solid tumors and have been life-saving for many patients (13, 25–35). However, CRS caused by CAR T cells is a major limiting factor in the successful utilization of cellular immunotherapy due to the competing risk of morbidity and mortality from the treatment itself (14). In order to improve CAR-based therapies, it is important to understand the limitations of the current CRS treatment approach and explore new strategies of CRS treatment and prevention.

### CAR Therapy—Structure Impacts Cytokine Production

Worldwide, well-over 500 active clinical trials are registered for CAR-based therapies, and the list continues to grow^1^. In general, autologous (or more rarely, allogeneic) T cells are transduced with retroviral, lentiviral, or transposon-based systems with the CAR construct. NK cells are also being investigated as viable alternatives to T cells (9, 36). While CD19 is the most utilized antigen in CAR trials to date, a variety of other antigens are now being investigated (37–46). Lymphodepletion with agents such as fludarabine and/or cyclophosphamide chemotherapy prior to CAR-T infusions provides reduction of T regulatory cells which can inhibit CAR-T cell activity and promotes expansion of these CAR-T cells. The use of lymphodepleting chemotherapy is now standard in clinical trials. This chemotherapy-enhanced expansion of engineered CAR-T cells can provoke initial cytokine secretion. The secondary effects of these activated T cells can later produce another wave of cytokine production by activating other surrounding immune and antigen-presenting cells, which will be discussed later (47, 48).

CARs are molecules synthetically designed to comprise an extracellular single-chain variable fragment, scFv (variable light chain, V_L_, and variable heavy chain, V_H_), from an antigen-specific antibody. This antigen-specific antibody allows CAR T cells to recognize tumor cells in a T-cell receptor (TCR) and human leukocyte antigen (HLA)-independent manner. In regard to CD19 as an extracellular receptor on CARs, since it is expressed throughout B cell maturation starting from the pro-B cell stage, targeting CD19 facilitates its use against different B cell tumors that have originated at distinct developmental stages. The cytoplasmic tail of CD3ζ is added as the primary signaling module, and in some cases, two out of four tyrosines within the immunoreceptor tyrosine-based inhibition motifs are mutated to optimize the signaling threshold. The extracellular domains are joined to intracellular signaling modules via hinge and transmembrane regions. The intracellular domains contain both the obligatory signaling module (CD3ζ) and the cytoplasmic tail of co-stimulatory molecules such as CD28 or CD137 (4-1BB). At a cellular level, combining the cytoplasmic tails of CD3ζ with CD28 or CD137 provides strong activating signals along with robust survival and proliferation signals. Early phase studies with CD19 CARs have demonstrated the utility of including these co-stimulatory signaling molecules in the CAR product (49–51).

CD3ζ uses ZAP-70/LAT/PLC-γ1 to activate NFAT or NF-κB pathways via augmenting Ca^2+^ and diacylglycerol (DAG) (52). Also, CD3ζ activates AP-1 through the Ras/MAPK pathway. CD28 and CD137 are also known to regulate overlapping and distinct signaling pathways (53, 54). Both CD28 and CD137 can regulate proliferation, survival, differentiation, and effector functions in CAR T cells by activating the PI(3)K/AKT/Bcl-X_L_ cascade (55). CD28 can also affect T cell proliferation and function through Grb2, FLNa, and Lck pathways (56, 57). Similarly, 4-1BB signaling is dependent on TNF-receptor Associated Factor, or TRAF pathway. Thus, the interactions of these multiple downstream signaling pathways from upstream inducible costimulatory molecules could be important keys to understanding how CAR-T cells produce cytokines at supraphysiologic levels.

Ultimately, the transduced CAR helps these engineered T cells to mediate a redirected response toward antigen positive tumor cells, which involves both granzyme-B and perforin for tumor lysis as well as generation of inflammatory cytokines and chemokines. The outcome of these signaling pathways in CAR T cells can provide effective anti-tumor cytotoxicity and enhance the production of inflammatory cytokines. Unique signaling pathways that exclusively regulate each of these functions are being defined.

### Cytokine-Release Syndrome: Pathogenesis of Cytokine Production

T cells play a pivotal role in tumor immunosurveillance (58). Effector functions of T cells correlate with beneficial graft-vs.-tumor responses (59). However, they are also the primary producers of pro-inflammatory cytokines and chemokines (60). Inflammation is an essential component of effector T cell mediated immune responses. However, acute inflammation at enhanced levels is highly destructive.

Antibody-based CAR receptors possess 10 to 60-fold higher affinity than the average affinity of TCR. An intrinsic dissociation constant of an affinity matured antibody can exhibit a *K*_d_ 5 × 10^−14^ compared to a CD8^+^ T cell-derived TCR that has a *K*_d_ 10^−7^. While TCR-engineered T cells can also potently engage cancer targets and have been shown to cause significant toxicity, there appear to be interactive antigen differences between a CAR and a TCR (61–63). Accordingly, the potency of CAR T cells is increased significantly compared to native T cells. While the extraordinary affinity of the CAR to its cognate antigen is the basis for augmented tumor killing, it also causes significant toxicity through supraphysiologic stimulation and cytokine production (49, 64, 65).

While cytokine production can occur physiologically during severe infections and graft-vs.-host disease, CRS is a complex clinical phenomenon characterized by the high activation of immune cells and immense production of proinflammatory cytokines (11, 14–16, 65–68). Acute CRS generally begins hours to days after CAR T cell infusion. In some cases, late CRS has appeared in patients 1–4 weeks after infusion, at a time when there is a significant CAR T cell expansion (47). Patients with CRS experience symptoms associated with this elevated amount of proinflammatory cytokines, including IFN-γ, IL-2, TNF-α, MIP-1, and GM-CSF (10, 50, 69). In addition to cytokines that originate from the CAR T cells themselves, additional cytokines (including IL-6, IL-8, and IL-10) primarily generated by bystander cells and professional antigen presenting cells, are also significantly elevated during CRS (32, 50, 70). This accounts for a secondary wave of cytokine production, which can often be higher than what is directly produced from CAR-T cells (32, 47, 48). Notably, this secondary burst of cytokines is associated with other hyperactive immune disorders such as hemophagocytic lymphohistiocytosis (HLH) and macrophage activation syndrome (MAS), with inflammatory markers such as C Reactive protein and ferritin becoming elevated (32).

The role of macrophages and other myeloid cells in the development of CRS has been confirmed through not only clinical assays and patient samples, but also in animal models. Giavridis et al. established that activated CAR T cells can recruit and activate macrophages and other myeloid cells and are the main source of IL-6. Authors also confirmed the important role of IL-1 blockade in improving symptoms and suggested that its ability to cross the blood brain barrier made it an ideal pharmacological candidate compared to tocilizumab (71). Similarly, Norelli et al. also confirmed though their preclinical animal studies that IL-1 was critically important in the pathogenesis of CRS, and also suggested that Anakira be considered as part of CRS therapy (72).

### Clinical Manifestations of CRS

CRS begins with mild symptoms, but it can quickly progress to life-threatening complications (13, 14, 26, 31, 35, 73–76). Multiple organ systems are involved in all phases. Fevers are an obligatory sign of CRS and typically precedes any other manifestation. Constitutionally, in addition to fevers, patients can develop rigors, nausea, and arthralgias. Hematologically, a picture consistent with disseminated intravascular coagulation can be seen, consisting of coagulopathy and hemorrhage. B cell aplasia is very common and a result of on-target-off-tumor effects. Gastrointestinal toxicities include colitis with diarrhea and abdominal pain and hepatitis. Cardiovascular side effects often prompt transfer to critical care settings, where tachycardia and hypotension can be supported with fluids and pressors. Other organ systems, including renal and pulmonary, can be affected by edema and third spacing, causing hypoxia and respiratory decompensation requiring intubation and ventilatory support. The neurological toxicities (CRES/ICANS) are some of the most puzzling and disturbing complications seen (77). While some patients can develop mild symptoms of headache and confusion, others have progressed (and in some cases rapidly) to seizures, cerebral edema, and death. Aphasia has also been identified as part of the neurotoxicity profile identified with CRES/ICANS. While some symptoms can occur during the CRS period, this has not been consistently seen, and CRES/ICANS can even occur days or weeks later (78). Table 1 highlights a summary of CAR-T trials with focus on CRS grade results (14, 17, 19, 20, 22, 42, 46, 79–82). The degree of CRS and CRES/ICANS have appeared to correlate in most cases with the use of preceding lymphodepletion, leukemia burden, and CAR-T cell dose, although it is still not completely clear why some patients do not develop any CRS and/or CRES/ICANS. Identifying biomarkers for use in CRS prediction algorithms is an emerging area of investigation to help guide patient risk, and if combined with patient characteristics, may become a helpful tool to determine CRS and CRES/ICANS risk (32).

**Table 1:** Summary of published CAR-T trials with focus on CRS outcomes.

**References**	**Target**	**Lymphodepletion**	**CRS Scale**	**Total patients treated**	**CRS Grade** **1 or 2**	**CRS** **Grade 3**	**CRS** **Grade 4**	**CRS** **Grade 5**	**Additional comments**
Lee et al. (14)	CD19	Fludarabine/ Cyclophosphamide (Flu/Cy)	Trial specific	21	10	3	3	0	4 patients received tocilizumab and/or steroids
Gardner et al. (22)	CD19	Flu/Cy (*n* = 14) No lymphodepletion (*n* = 31)	Trial specific	45	33	10			16 patients received tocilizumab; 10 received steroids
Neelapu et al. (20)	CD19	Flu/Cy	Lee	101	82	9	3	1	Grade 5 event was due to hemophagocytic lymphohistiocytosis. 43 patients received tocilizumab and 27 received steroids
O'Rourke et al. (79)	EGFRvIII	None	Not described	10		0			
Ramos et al. (46)	CD30	None	Not described	9		0			
Schuster et al. (19)	CD19	Cy-only (*n* = 10) Bendamustine (*n* = 8) RT + Cy (*n* = 4) Other (*n* = 6)	Penn	28	11	4	1	0	1 patient received tocilizumab; none received steroids
Fry et al. (42)	CD22	Flu/Cy	Lee	21	16	0	0	0	
Maude et al. (17)	CD19	Flu/Cy (*n* = 71) Cytarabine/Etoposide (*n* = 1)	Penn	75	23	16	19	0	28 patients received tocilizumab
Park et al. (18)	CD19	Cy (*n* = 43) Flu/Cy (*n* = 10)	MSKCC	53	31	13		1	23 patients received tocilizumab and/or steroids
Bishop et al. (80)	CD19	Flu/Cy or bendamustine	Penn	7	2	2	0	0	
Cao et al. (81)	CD19	Flu/Cy	Lee	11	3	6	0	0	All patients received anti-PD-1 ab therapy 3 days after CAR T cells. None required tocilizumab or steroids
Zhao et al. (82)	BCMA	Cy	Lee	57	47	4	0	0	24 patients received tocilizumab.

### Treatment for CRS Includes Pharmacological Interventions and Supportive Care: The Pros and Cons of This Approach

Currently, immunosuppression is the primary therapeutic approach to treat life-threatening complications of CRS. With the concurrent development of distinct CAR-T constructs and clinical trials across different research groups, numerous CRS grading scales have developed over the years [Table 2; (31, 78, 83–86)]. While these scales are similar in the fact that they begin with mild CRS symptoms (grade I) and end in death (grade V), they differ in how they progress between the different grades. This makes it challenging to compare CRS results across multiple trials. New consensus workshops have identified a uniform method that will likely be adopted when considering future CRS reporting, although in the interim, it is important to look closely at the grading scale used when comparing the safety results across trials (85).

**Table 2:** CRS definitions across different scales.

**Scale**	**Grade I**	**Grade 2**	**Grade 3**	**Grade 4**	**Grade 5**
MSKCC (83)	Mild symptoms requiring observation or supportive care only	• Hypotension requiring any vasopressors <24 h • Hypoxia/dyspnea requiring O_2_ <40%	• Hypotension requiring any vasopressors ≥24 h • Hypoxia/dyspnea requiring O_2_ ≥40%	• Hypotension refractory to high-dose vasopressors • Hypoxia/dyspnea requiring mechanical ventilation	Death
Lee et al. (31)	Fever, constitutional symptoms	• Hypotension responsive to fluids or one low dose pressor • Hypoxia responsive to <40% O_2_ • Organ toxicity: grade 2	• Hypotension requiring multiple pressors or high dose pressors • Hypoxia requiring ≥40% O_2_ • Organ toxicity: grade 3, grade 4 transaminitis	• Hypoxia requiring mechanical ventilation • Organ toxicity: grade 4, excluding transaminitis	Death
Penn (84)	Mild reaction	• Organ toxicity: grade 2 creatinine, grade 3 transaminitis • Hospitalization for management of CRS-related symptoms	• Organ toxicity: organ dysfunction requiring hospitalization, including grade 4 transaminitis or grade 3 creatinine • Hypotension requiring IV fluids or low-dose vasopressors • Coagulopathy requiring blood product transfusions • Hypoxia requiring supplemental oxygen	• Hypoxia requiring mechanical ventilation • Hypotension requiring high-dose vasopressors	Death
CARTOX (78)	• Temperature ≥38°C • Grade 1 organ toxicity	• Hypotension responsive to IV fluids or low-dose vasopressors • Hypoxia requiring FiO_2_ <40% • Grade 2 organ toxicity	• Hypotension needing high-dose or multiple vasopressors • Hypoxia requiring FiO_2_ ≥40% • Grade 3 organ toxicity or grade 4 transaminitis	• Life-threatening hypotension needing ventilator support • Grade 4 organ toxicity except grade 4 transaminitis	Death
ASTCT (85)	Fever without hypotension or hypoxia	Fever with either: • Hypotension not requiring vasopressors • Hypoxia requiring low-flow nasal cannula or blow-by	Fever with either: • Hypotension requiring a vasopressor • Hypoxia requiring high-flow nasal cannula, facemask, non-rebreather mask, or Venturi mask	Fever with either: • Hypotension requiring multiple vasopressors (excluding vasopressin) • Hypoxia requiring positive pressure ventilation	Death
CTCAE5.0 (86)	Fever with or without constitutional symptoms	Hypotension responding to fluids; hypoxia responding to <40% O_2_	Hypotension managed with one pressor; hypoxia requiring ≥ 40% O_2_	Life-threatening consequences; urgent intervention indicated	Death

When identified early, CRS can be managed with supportive care ± anti-IL-6R mAb (tocilizumab) (13, 14, 87). Patients who respond to tocilizumab resolve CRS within a few hours to days; however, there are others who do not respond to this monoclonal antibody and require the administration of corticosteroids (31). With experience gained in identifying, classifying, and treating CRS early, outcomes have improved greatly with proactive supportive care and pre-emptive pharmacological support. Furthermore, tocilizumab was approved for treatment of CRS in August 2017, easing its ability to be accepted more uniformly as a standard of care treatment for CRS (88).

However, there are several concerns with using pharmacological agents to treat CRS. First, there is some concern that systemic immunosuppression caused by these drugs may diminish the efficacy of CAR-T cells. Furthermore, dampening the immune system may make patients who are already sick and compromised from lymphodepleting chemotherapy more prone to infections. Furthermore, with more studies focused on decreased persistence of CAR-T, it is unclear if dampening their response with immune suppressive therapies will ultimately affect their ability to persist in order to be a true “living” biological drug in patients (89). Finally, understanding the biochemistry of CRS brings to light that targeting the IL-6 receptor with tocilizumab does not always work for CRES, and by the time a direct Il-6 antagonist such as siltuximab is used, it may be too late in the process to have a substantial impact. Many have speculated that use of many of these agents, including anti IL-1, may be targeting cytokines that are further downstream from the instigating events that begin this cascade of inflammation. Thus, waiting for CRS/CRES to occur and using agents to neutralize downstream cytokines may not be the best strategy to consider. While some have advocated for pre-emptive treatment with agents such as tocilizumab to prevent severe CRS, this is still under investigation (90).

Perhaps most importantly, the outcome of CRS can be life-threatening, and there have been a number of patient deaths following treatment with CAR-T cells (16). Because current algorithms are waiting until CRS symptoms occur before treating, patients are being put at risk. Therefore, novel approaches are needed to manage and prevent CRS. Because CAR's are inherently engineered cellular products, designing a safer CAR could be one approach to consider.

### Engineering Safer CAR Products: Is It Possible?

With the advent of clustered regularly interspaced short palindromic repeats (CRISPR) technology, it is now possible to engineer a CAR-T that may have different and more specifically-controlled properties than virally-transduced cells. Specifically, it has been shown that targeting a CAR-coding sequence to the T cell receptor (TCR) locus may prevent accelerated T-cell exhaustion by decreasing tonic activation and TCR-induced autoimmunity. This decreased tonic activation may also decrease CRS (91). Another method that directs the engineered CAR to operate through the native TCR is a T-cell antigen coupler, or TAC (92). The TAC has three components, including an antigen-binding domain, a TCR-recruitment domain, and a co-receptor domain, for a more controlled design that can also decrease off-target toxicity. Another method that has been explored is the use of synthetic Notch receptors to induce T cell response in a customizable manner. Specifically, these receptors, when engaged to its cognate antigen, induce a transmembrane cleavage that releases the intracellular transcriptional domain to penetrate the nucleus and activate a synthetic response (93). While these constructs are using the newest technologies for cellular engineering, they are still in the early phases of development. Finally, another method to consider is to decipher the downstream signaling pathways that become activated once a CAR is engaged with its cognate ligand. By targeting the activation of these pathways that produce excessive cytokines but maintain cytotoxic potential, there is strong potential to regulate CRS (54, 66).

### The FynADAP Pathway: Can It Be Targeted to Develop a Safer CAR?

**Figure 1:**
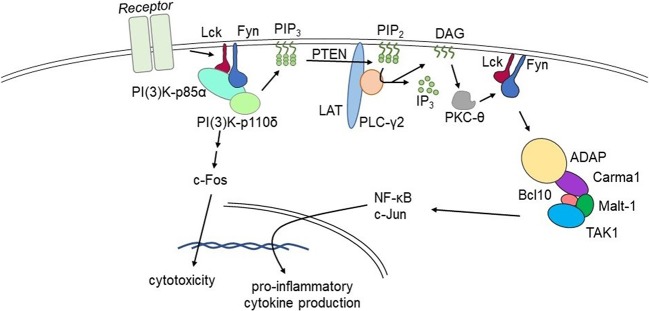
Fyn activates divergent signaling cascades in effector lymphocytes. Signaling via the Lck→Fyn→ADAP→CARMA1→Bcl10 pathway that is obligatory for the production of inflammatory cytokines. Divergent signaling via the Lck→Fyn→PI(3)K pathway primarily facilitates cell-mediated cytotoxicity. Lack of ADAP significantly reduces pro-inflammatory cytokine production without affecting cell-mediated cytotoxicity in pre-clinical models.

Our lab is focused on NK cell immunobiology, and NK cell intracellular signaling pathways are directly applicable to understanding T cell functionality (66). Our work has shown that adhesion and degranulation-promoting adapter protein (ADAP) serves as a positive regulator of proinflammatory cytokine production (54). The FynADAP complex exclusively regulates the production of inflammatory cytokines (94). Most importantly, lack of ADAP does not affect the NK cell-mediated anti-tumor cytotoxicity. These findings establish ADAP as a potential molecular target to reduce the production of inflammatory cytokine and chemokine production. This 130 kDa protein is expressed in multiple cell types including NK and T cells. It functions as a connecting link between the upstream Fyn and downstream signaling proteins Carma1 and TAK1. ADAP also interacts with SLP76 and SKAP55. The potential interaction sites of these signaling proteins in ADAP have been largely defined. Recently, as shown in Figure 1, we defined a Lck → Fyn → ADAP → CARMA1 → Bcl10 pathway that is obligatory for the production of inflammatory cytokines (54). Lack of ADAP in T or NK cells significantly reduces the production of IFN-γ, GM-CSF, TNF-α, MIP-1α, MIP-1β, and RANTES; however, the anti-tumor cytotoxicity was intact. In both T and NK cells, ADAP plays an essential role in immunoreceptor tyrosine-based activation motif-dependent receptor activation and is involved in activation integrins including LFA1. Loss of ADAP in T cells decreases their proliferation and cytokine production efficiency in response to limiting antigen doses (95).

These findings provide a feasible clinical approach to reduce the production of inflammatory cytokines and chemokines, and thereby the severity of CRS. Stimulation through TCR and CD28 utilizes ADAP to facilitate signaling downstream of the Carma1-Bcl10-Malt1 (CBM) complex, which leads to phosphorylation and degradation of IκBα and nuclear translocation of NF-κB (96). While the molecular mechanism whereby ADAP regulates the formation of the CBM has not been fully elucidated, the essential function of ADAP in linking CBM via Carma1 to PKC-θ is well-documented (97). NF-κB, which is sequestered in the cytosol through binding to IκBα, translocates into the nucleus (97). Carma1 plays an obligatory role in the nuclear translocation of NF-κB following activation of T or NK cells (98, 99). In addition to interactions with Carma1, ADAP also recruits TAK1, which facilitates the phosphorylation of IKKα and IKKβ, components of NF-κB signaling pathway. In T cells, ADAP contributes to CBM complex formation in response to ITAM-containing receptors (96, 97, 99–101). Thus, targeting ADAP in T cells could help to selectively attenuate cytokine production, without reducing cytotoxicity. The feasible approaches to target ADAP include CRISPR-CAS9-based deletion of ADAP in CAR-transduced T and NK cells, small molecule-based interference of interactions in the Fyn-ADAP-CBM pathway, and utilization of small hairpin interfering RNAs.

There are three major concerns with this approach that need to be addressed prior to blocking the interaction of Fyn and ADAP in clinical trials. (1) Cytokines such as IFN-γ are obligatory to clear certain types of malignancies. In general, cytokines and chemokines play a central role in orchestrating a productive anti-cancer response. Therefore, strategies to reduce the production of inflammatory cytokines should not completely curtail overall cytokine production, which would likely negatively impact anti-tumor cytotoxicity. (2) Cytokines are required for CAR-T homeostasis and likely survival and persistence. While curtailing cytokine production will not eliminate their production entirely, it is unclear to what degree of innate cytokine needs are necessary to maintain CAR-T perseverance. (3) As discussed earlier, cytokines responsible for CRS likely come from two sources. The primary source is the CAR T cells themselves that initiate the first wave of proinflammatory cytokine production, which this approach should help control. However, the secondary wave of cytokines, such as increased IL-6 production, originate from myeloid cells in response to augmented cytokine signaling from CAR T cells and native effector lymphocytes. In theory, reducing the levels of proinflammatory cytokines generated by CAR T cells should help to contain both the primary as well as indirectly, the secondary waves of cytokine productions.

## Summary and Future Directions

CAR-T immunotherapy has changed the landscape of cancer treatment. However, CRS occurs in two-thirds of patients, and in its worst form can lead to death. While tocilizumab can be effective in treating CRS, once it occurs, CRS development may lead to increased morbidity, hospitalization, and cost, and may limit the dose of CAR T cells that can be used clinically. And while current algorithms have enhanced identification and treatment for CRS to decrease mortality, using these pharmacological interventions pre-emptively has not yet been established as a standard of care. Therefore, knowledge of the signaling pathways that uniquely regulate anti-tumor cytotoxicity and inflammation is critical in identifying potential novel targets for containing CRS. Our recent work evaluating the FynADAP pathway provides an archetypical model to validate blocking these unique signaling pathways to contain cytokine production as one method to engineer a safer CAR-T cells. Successful translation of this and other engineered strategies to reduce CRS in this intrinsic manner is a compelling approach to this important problem.

## Author Contributions

MT, TK, and SM wrote and edited this manuscript.

### Conflict of Interest

The authors declare that the research was conducted in the absence of any commercial or financial relationships that could be construed as a potential conflict of interest.

## Footnotes

^1^https://www.clinicaltrials.gov (cited July 10, 2019).
